# Growth and physiological response of *Kandelia obovata* and *Bruguiera sexangula* seedlings to aluminum stress

**DOI:** 10.1007/s11356-021-17926-0

**Published:** 2022-01-29

**Authors:** Li Ma, Shengchang Yang

**Affiliations:** 1 Key Laboratory of the Coastal and Wetland Ecosystem (Ministry of Education), College of the Environment and Ecology, Xiamen University, Xiamen, China; 2 Department of Chemical Engineering, Chengde Petroleum College, Chengde, China

**Keywords:** Aluminum, *Kandelia obovata*, *Bruguiera sexangula*, Antioxidant enzyme, Metal element

## Abstract

The role of mangroves as a biogeochemical buffer for heavy metal pollutants in coastal wetlands has been demonstrated, but knowledge gaps still exist on the tolerant capacity of mangroves to aluminum (Al). This study assessed the growth and physiological response of viviparous mangroves *Kandelia obovata* and *Bruguiera sexangula* to Al stress. The two mangrove seedlings were treated with AlCl_3_ at concentrations of 0 (as control) to 100 mmol L^−1^, and the impact of Al on their growth and antioxidant parameters were determined. Additionally, the accumulation and translocation of metal elements were estimated in *B. sexangula* seedlings under relative long-term Al stress. *K. obovata* appeared to survive with a tolerance potential of 10 mmol L^−1^ AlCl_3_, whereas *B. sexangula* had a higher tolerant ability of 50 mmol L^−1^ AlCl_3_. Both root elongation and seedling growth were inhibited by Al stress. The exposure to 25–100 mmol L^−1^ AlCl_3_ induced increases in membrane lipid peroxidation and osmoprotectant molecule (proline) in mangrove seedlings. Both mangrove seedlings revealed significant changes in antioxidant enzyme activities that were attributed to Al stress-induced oxidative damages. The activities of superoxide dismutase, catalase, peroxidase, and/or ascorbate peroxidase were differently impacted by the treatment time (7 days for short term *versus* 60 days for long term) and AlCl_3_ concentrations in *K. obovata* and *B. sexangula* seedlings. For *B. sexangula* seedlings, Al accumulation was in an order root > leaf > stem, whereas the translocation of metal elements in the aboveground tissues (leaf and stem) was differently impacted by Al stress. In conclusion, this study provides insights into different Al-tolerant abilities operated in two mangrove species that are widespread in coastal wetlands of China.

## Introduction

Anthropogenic activity has resulted in increases in the carbon dioxide (CO_2_) release and the global atmospheric CO_2_ concentration in the past two centuries (Siegenthaler et al. 2005; Le Quéré et al. 2015; NOAA/ESRL 2019). A large proportion of excessive CO_2_ dissolved in the ocean and coastal waters is causing acidification (Sabine et al. 2004; Doney et al. 2009; Cai et al. 2011; Garilli et al. 2015; Kwiatkowski et al. 2016; USEPA 2020). As a special type of tropical forests growing in tidal flooded coastal areas, mangrove forests are salt-tolerant and submerge-resistant (Ball et al. 1997; Chen and Wang 2017), and their unique distribution at the transition zone between terrestrial and aquatic environments makes them easily impacted by costal acidification (Sippo et al. 2016). Mangrove soils are neutral to slightly acidic due to the sulfur-reducing bacteria, and the acid sulfate soils are rich in aluminum oxide (Al_2_O_3_) (Sparks 2003; Alongi 2005; Blake 2005; Bleam 2012). Because the amounts of dissolved aluminum cations (Al^3+^) are significantly enhanced and released compared with other heavy metals, high concentrations of mobile aluminum (Al) has become a potential pressure for plants (Cook et al. 2000; Pred and Cox 2002; Ramos e Silva et al. 2006).

Previous studies have demonstrated that mangrove seedlings are able to grow under very high concentrations of heavy metals (Walsh et al. 1979; MacFarlane and Burchett 2002; Miao et al. 2007; Ravikumar et al. 2007; Dai et al. 2017) and salt stress (Takemura et al. 2000; Wang et al. 2014; Xing et al. 2019), although with negative effects on the growth. Mangroves have a high accumulation capability of metals, which can retain and provide a biogeochemical buffer for heavy metal pollutants (Tam 1998; Jones et al. 2000; MacFarlane and Burchett 2000; MacFarlane et al. 2003; Ramos e Silva et al. 2006; MacFarlane et al. 2007; Zhou et al. 2010). Mangroves are highly capable of uptake of metals and nutrients via root system from coastal sediments (MacFarlane et al. 2007; Marchand et al. 2016), which provides them a key role in phytoremediation (Agoramoorthy et al. 2008). Mature mangroves are known to be a particular ecosystem that can buffer coastal acidification (Sippo et al. 2016), which may be categorized as Al accumulator plants (Kombo et al. 2005; Isyrini 2014). Yet little is known regarding the effects of Al on mangroves, particularly under acidic environments. To our knowledge, no research has confirmed whether mangrove seedlings, when grown under high concentrations of Al, behave the same as existing mature mangroves. Thus, the evaluation on the adaptation capability of mangrove seedlings is important. Until now, the impact of Al on the storage and transport of other major and trace elements, classified as macro-nutrients (Na, K, Mg, and Ca) and trace metals (Mn, Fe, Cu, and Zn), respectively, in the mangrove seedlings remains unknown.

Aluminum adversely affects the germination of seeds and plant growth, such as inhibition of root elongation, seedling chlorosis, changing enzyme activities in various metabolic pathways, and affecting synthesis of proline and novel proteins (Samac and Tesfaye 2003; Tamás et al. 2003; Sivaguru et al. 2003; Fukuda et al. 2007; Chandra and Keshavkant 2021). It has been demonstrated that the formation of reactive oxygen species (ROS), such as hydrogen peroxide (H_2_O_2_), superoxide (O_2_^−^), and hydroxyl (OH) radicals, is stimulated by Al stress (Nahar et al. 2016; Al Mahmud et al. 2019; Devi et al. 2020). In order to protect cells from excessive ROS stress, antioxidant enzymes including superoxide dismutase (SOD), catalase (CAT), peroxidases (POD), and ascorbate peroxidase (APX) are induced to avoid oxidative damages (Tam and Wong 1996; MacFarlane and Burchett 2001; Zhang et al. 2007; Caregnato et al. 2008; Liu et al. 2009; Huang and Wang 2010; Wei et al. 2010). However, information on the growth response to Al stress and its induction of oxidative stress is scarce in mangrove seedlings. Based on previous research results that indicate that mangrove seedlings are able to grow under very high concentrations of heavy metals and salt stress, we hypothesized that similar tolerance and adaptive characteristics may occur in mangrove seedlings to high Al concentrations: (1) mangrove seedlings exposed to acidic conditions with elevated Al concentrations can survive, but with some disruptions in seedling growth, and (2) antioxidant enzymes in seedlings are activated to defend Al-induced oxidative stress during natural growth.

Therefore, in order to elucidate the effects of Al stress on the growth and adaptation capability of mangrove seedlings, a laboratory study was conducted by culturing propagules of two natural viviparous mangroves and subjecting them to a range of aluminum chloride (AlCl_3_) treatments. *Kandelia obovata* (S., L.) Yong and *Bruguiera sexangula* (L.) Poir. are two main mangroves belonging to salt-excluding species (i.e., species that exclude salt at the roots), which are of high biomass among mangroves in the costal wetland ecosystem of China (Chen et al. 2009; Ye et al. 2013) and are well tolerant of adverse environmental conditions (Lu et al. 2012; Chen et al. 2017). The main objectives of this study are (1) to evaluate the response of the two viviparous mangrove seedlings to Al stress, including their growth, lipid peroxidation levels, and osmotic stress (indicated by proline contents), especially the activities of antioxidant enzymes involved in ROS scavenging; and (2) to determine the accumulation and translocation of metal elements within tissues of mangrove seedlings after relative long-term Al stress treatments.

## Materials and methods

### The sampling area

The sampling site for mangrove propagules is characterized with good water quality, non-metal pollution, and non-acid sulfate soil. Mature *K. obovata* propagules were collected from the Mangrove Nature Reserves located in the Jiulong River estuary in Fugong Town (24° 26′ N, 117° 54′ E), Longhai County, Zhangzhou City, Fujian Province, China. The area belongs to the southern subtropical maritime climate, with a mean annual air temperature of 21.0 °C (Ye et al. 2013) and annual precipitation of 1,450 mm. Regular semi-diurnal tides with an average salinity of water is 17.1 psu (Chen et al. 2007). The sediment in this area is primarily composed of silt and sand with pH values of 5.5–7.1 (Li and Ye 2014). Mature *B. sexangula* propagules were collected from the Mangrove Nature Reserves located in the Qinglan harbor, Wenchang River estuary (19° 34′ N, 110° 24′ E), Wenchang City, Hainan Province, China. The mean annual temperature and precipitation at the site are 24.3 °C and 1,975 mm, respectively, with a rainy season between May and October. Irregular diurnal tides with an average salinity of water are > 30 psu. The pH value of the near shore soils ranges from 4.9–6.2.

### Experiments

#### Propagation and germination

The laboratory study was conducted in an indoor nursery at the Key Laboratory of the Coastal and Wetland Ecosystem (Ministry of Education), Xiamen University, China. Healthy and mature *K. obovata* propagules with lengths of 20.80 ± 0.59 cm and fresh weights (FWs) of 12.17 ± 1.69 g and *B. sexangula* propagules with lengths of 8.23 ± 1.04 cm and FWs of 12.20 ± 0.68 g were randomly planted in the plastic sifters with pots, with 5 propagules in each sifter. Each sifter and pot were filled with 2.5 kg of washed river sand (diameter: 2–4 mm). Artificial seawater containing Hoagland’s culture solution was used for propagation and treatments, with a salinity of 3.0 psu by dissolving 85.5 mM NaCl. This salinity level is considered suitable for the development of mangroves that are grown in a nursery environment (Downton 1982; Takemura et al. 2000).

#### Al Treatment set-up

After 1 month of natural growth, *K. obovata* and *B. sexangula* seedlings were treated by Al in the form of AlCl_3_ solution. The selection of chloride (Cl) metals was based on the consideration that this form is tolerable by mangroves, therefore reducing the toxicity of the Cl ions (salt stress) (Burchett et al. 1984; Takemura et al. 2000; Parida et al. 2004; Yan and Chen 2007; Wang et al. 2014; Hossain et al. 2017; Basyuni et al. 2018). Due to little is known regarding the effects of Al on mangrove seedlings, the chosen concentrations of AlCl_3_ were primarily based on the Al limit reported on representative terrestrial plants in previous studies (Cakmak and Horst 1991; Darkó et al. 2004; Morita et al. 2008), and the tolerance range to heavy metals (Walsh et al. 1979; MacFarlane and Burchett 2002; Miao et al. 2007; Ravikumar et al. 2007; Dai et al. 2017) and salt stress (NaCl) on laboratory-grown mangrove seedlings (Takemura et al. 2000; Wang et al. 2014; Xing et al. 2019).

We designed AlCl_3_ treatment experiments using a relative lower concentration of 0–1.0 mmol L^−1^ and a relative higher concentration of 0–100 mmol L^−1^. The AlCl_3_ treatments at 0–1.0 mmol L^−1^ were applied to *K. obovata* seedlings, whereas AlCl_3_ treatments with a concentration gradient of 0 (as control), 10, 25, 50, 75, and 100 mmol L^−1^ were applied to both *K. obovata* and *B. sexangula* seedlings. AlCl_3_ solution with a final pH value of 4.2 was added to each pot in each treatment, with four replicates for each treatment. Culture solutions were renewed weekly to maintain a relatively constant AlCl_3_ concentration during the experimental period. All cultivations were kept in an indoor nursery with an air temperature of 25–32 °C. The duration of the treatment was up to 7–90 days, depending on the mortality of the seedlings. Additionally, the short-term (168 h = 7 days) AlCl_3_ treatments at concentrations of 25 and 100 mmol L^−1^ were applied to *K. obovata* seedlings to estimate the effect of Al on lipid peroxidation and antioxidant enzyme activities*.*

### Parameter analysis

#### Growth parameters

Growth measurements from each set of seedlings included apparent healthy conditions, survival of seedlings, the height of seedling, and the average root length. The stem height of seedlings was measured from the top of the propagules where the stem emerged to the bottom of the most distal opened pairs of leaves. The root length increment (final root length minus initial root length) was measured to evaluate Al-induced root-growth inhibition.

#### Lipid peroxidation and osmotic stress parameters

The level of lipid peroxidation that was expressed as malondialdehyde (MDA) content was evaluated by the thiobarbituric assay described by Shah et al. (2001) with some modifications. Briefly, 1.0 g of fresh leaf or root was homogenized in 10 mL of phosphate buffer (pH: 7.8) on an ice bath and centrifuged at 15,000 g and 4 °C for 20 min. One milliliter of supernatant was mixed with 1.0 mL of 0.5% 2-thiobarbituric acid (TBA) in 10% trichloroacetic acid (TCA) (w/v) solution. After being incubated at 95 °C for 15 min, the reaction was terminated by cooling and the mixture was centrifuged at 4,000 rpm for 20 min. The absorbance was measured at 532 and 600 nm, and the non-specific absorbance at 600 nm was subtracted from the 532 nm absorbance. MDA contents were calculated by using an extinction coefficient (ε) of 155 mmol L^−1^ cm^−1^ and were expressed as nmol g^−1^ or mg g^−1^ of FW.

The osmotic stress indicated by free proline content was determined with a ninhydrin colorimetric assay according to Ábrahám et al. (2010). Plant materials were homogenized in 3% sulfosalicyclic acid (5 μL mg^−1^ of FW) on an ice bath and centrifuged at 11,500 g for 5 min. One hundred microliters of supernatant was mixed with 200 μL of glacial acetic acid and 200 μL of acidic ninhydrin. After incubating the mixture at 96 °C for 60 min followed by quick cooling, 1 mL of toluene was added and stirred for 30 s. After 5 min, the chromophore containing toluene was measured spectrophotometrically at 520 nm. The proline content was determined using a standard curve and calculated on the FW basis.

#### Enzyme extraction and estimation of antioxidant enzyme activities

Methods taken from references were optimized before being applied to *K. obovata* and *B. sexangula*. Enzyme extraction was prepared following the method of Gossett et al. (1994). Soluble protein contents were determined according to Bradford (1976) using bovine serum albumin (BSA, Sigma) as the standard. Absorbencies were measured with a UVICAM UV-300 (Thermo Spectronic, USA) spectrophotometer.

SOD activity was measured based on the method described by Dazy et al. (2009). The photochemical reduction of nitro blue tetrazolium (NBT) was inhibited and the level of inhibition was used to quantify SOD activity. The reaction mixture contained 100 μL of plant extract, 100 μL of methionine (130 mM), 100 μL of riboflavin (600 mM), 100 μL of NBT (22.5 mM), and 600 μL of 125 mM potassium–phosphate buffer (pH: 7.8). After 15 min of illumination reaction (ambient light), the absorbance was measured spectrophotometrically at 560 nm and 25 °C. Unit of SOD activity was defined as the amount of enzyme required to cause 50% inhibition of the rate of NBT reduction measured at 560 nm under experimental conditions.

POD activity was determined according to Yin et al. (2005) with modifications. The reaction mixture contained 100 μL of plant extract, 1.5 mL of 62.5 mM sodium phosphate buffer (pH: 7.8), and 20 mM 2-methoxyphenol. The reaction was initiated by adding 10 μL of hydrogen peroxide (H_2_O_2_) (100 mM) at 25 °C for 5 min. POD activity was evaluated by measuring the rate of increase in absorbance at 470 nm.

CAT activity was determined according to Chaoui et al. (1997) with modifications. The reaction mixture contained 100 μL of plant extract and 900 μL of 62.5 mM sodium phosphate buffer (pH: 7.8) with 100 mM H_2_O_2_. The reaction was initiated by adding 10 μL of H_2_O_2_ (100 mM) at 25 °C for 5 min. CAT activity was evaluated by measuring the rate of disappearance of H_2_O_2_ at 240 nm with a molar extinction coefficient (ε) of 36.6 mM^−1^ cm^−1^.

APX activity was determined according to Garcia-Limones et al. (2002). The reaction mixture contained 100 μL of plant extract, 100 μL of ascorbate solution (5 mM), 100 μL of H_2_O_2_ (100 mM), 100 μL of EDTA solution (1 mM), and 125 mM potassium–phosphate buffer (pH: 7.0). APX activity was evaluated by measuring the rate of ascorbate oxidation at 290 nm with an extinction coefficient (ε) of 2.8 mM^−1^ cm^−1^.

#### Metal element analysis

For metal element analysis, samples of the leaf, stem, and root were collected, dried overnight at 85 °C, and then ground into fine powder with a ball milling instrument. Using a microwave digestion system (MARS-240/50, CEM, USA), 0.1 g of each powder sample was digested with 4 mL of nitric acid (HNO_3_) and 1 mL of H_2_O_2_ (30%). One hour later, the solution was transferred to a capacity bottle and filled with ultrapure water (UPW) to 100 mL. Metal elements including Na, Mg, Al, K, Ca, Mn, Fe, Cu, and Zn in *B. sexangula* seedlings were measured by inductively coupled plasma-mass spectrometry (ICP-MS, PE, USA). Each sample was repeated 3–4 times.

### Statistical analysis

Mean and standard deviation (SD) values of replicate seedling samples under each experiment group were calculated. All data obtained were subjected to one-way analysis of variance (ANOVA) (SPSS 18.0 for single-factor ANOVA). Statistical significance was assigned at *P* < 0.05. The relationships between variables were determined via correlation analysis. All graphs were plotted using SigmaPlot (version 10.0).

The enrichment factor (EF) of metals in each tissue of mangrove seedlings (i.e., leaf, stem, and root) was used to evaluate the elemental enrichment of mangrove tissues from the environment (i.e., total metal in culture solution). The EF of metal (Al in the case of this study) was determined using the ratio of the concentration of Al in a given sample (mangrove tissues) relative to the AlCl_3_ substance in the culture solution (i.e., Al_sample_/Al_substance_) (Costa et al. 2020). The mobility of metal that controls the distribution and accumulation of metals in mangrove tissues (MacFarlane and Burchett 2002) was assessed by the translocation factor (TF). The TF was determined by the ratio of metal concentration between the aboveground tissue (i.e., leaf and stem) and the underground tissue (i.e., root) (MacFarlane et al. 2007).

## Results

### Growth response of *K. obovata* and *B. sexangula* seedlings to aluminum stress

**Fig. 1:**
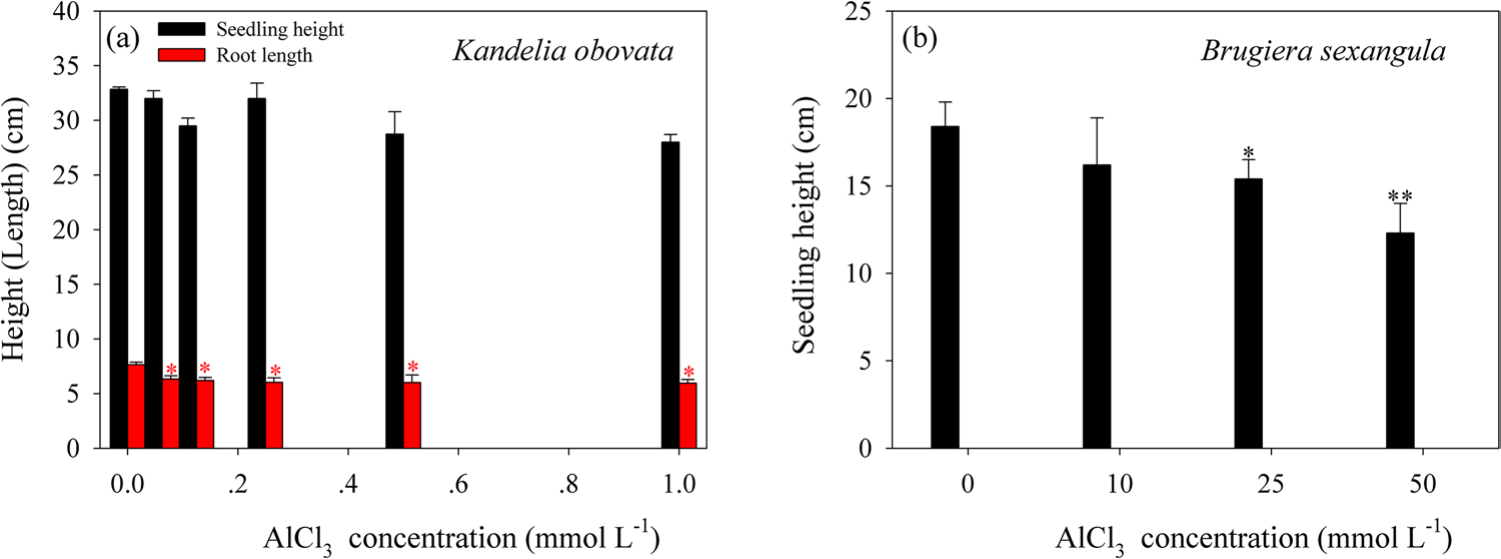
Growth response of *K. obovata* seedlings after long-term (90 days) treatments by low concentrations of AlCl_3_ (0–1.0 mmol L^−1^) (**a**), and growth response of *B. sexangula* seedlings that treated with 60 days by high concentrations of AlCl_3_ (0–50.0 mmol L^−1^) (**b**). Values are mean ± SD. “*” above the bar indicates a significant difference *versus* 0 mmol L^−1^ AlCl_3_ (control) at *P* < 0.05, and “**” above the bar indicates a significant difference *versus* 0 mmol L^−1^ AlCl_3_ (control) at *P* < 0.01

After relative long-term (90 days) treatments on *K. obovata* seedlings by low concentrations of AlCl_3_ (0.0625–1.0 mmol L^−1^), a significant decrease in average root length by 17.3–22.2% relative to that of control was observed, whereas there was no significant difference in seedling height (Fig. 1a). After being treated with high concentrations of AlCl_3_ (10–100 mmol L^−1^), the seedlings growing under 10 mmol L^−1^ AlCl_3_ showed no significant difference in growth conditions compared to that of control within 30 days. Whereas the natural growth of *K. obovata* seedlings was significantly inhibited by 25–100 mmol L^−1^ AlCl_3_ treatment, showing curled and wilted leaves with observed brown lesions, the number of new leaves also reduced 33.3–50.0% compared to that of control, the root tips turned black, and the whole roots became brittle. All (100%) of the seedlings were injured and eventually died within 7–15 days.

The natural growth of *B. sexangula* seedlings was significantly inhibited by 100 and 75 mmol L^−1^ AlCl_3_ treatments, and they finally died of root necrosis within 7 and 15 days, respectively. Under a AlCl_3_ concentration of 50 mmol L^−1^, the seedling growth became gradually stunted. Approximately 60 days later, a significant decrease in seedling height by 16.3% and 33.2% compared to that of control was observed for seedlings treated by 25 and 50 mmol L^−1^ AlCl_3_, respectively (Fig. 1b).

### MDA and proline contents in *K. obovata* and *B. sexangula* seedlings

**Fig. 2:**
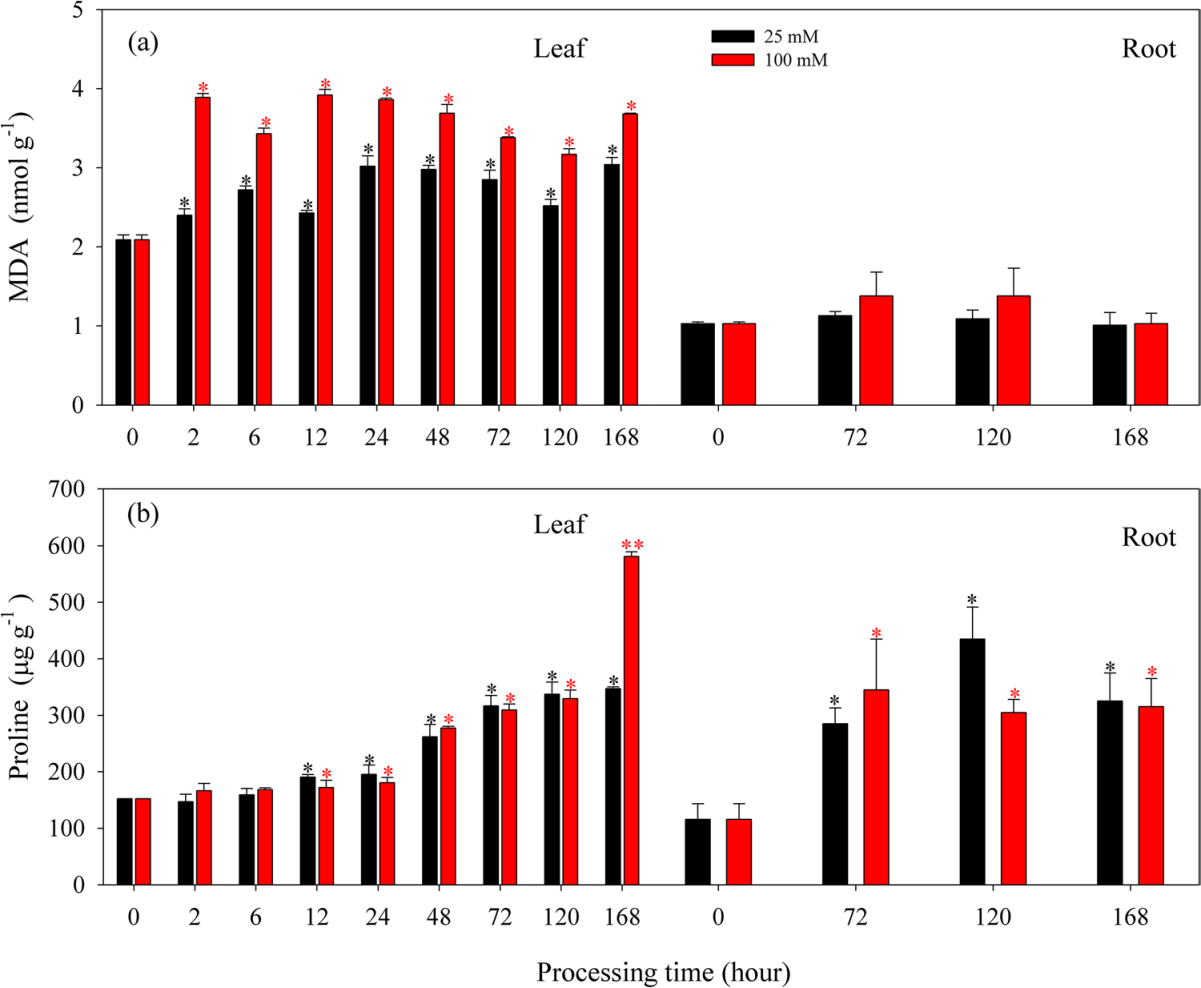
Changes in MDA content (**a**) and free proline content (**b**) in the leaf and root of *K. obovata* seedlings under short-term (168 h) treatments with 25 mmol L^−1^ (25 mM, black bar) and 100 mmol L^−1^ (100 mM, red bar) AlCl_3._ Values are mean ± SD. “*” above the bar indicates a significant difference *versus* 0 h group (control) at *P* < 0.05, and “**” above the bar indicates a significant difference *versus* 0 h group (control) at *P* < 0.01

The lipid peroxidation level (expressed as MDA content) and osmotic stress response (indicated by proline content) in *K. obovata* seedlings under short-term (168 h) treatments with 25 and 100 mmol L^−1^AlCl_3_ are presented in Fig. 2. The contents of MDA were significantly increased in the leaf after 2-h treatment under both AlCl_3_ concentrations (Fig. 2a), while there were no significant changes in MDA contents in the root (Fig. 2a). Overall, the MDA content was significantly higher after being stressed by 100 mmol L^−1^ AlCl_3_ than that by 25 mmol L^−1^ AlCl_3_ (*P* < 0.05). The proline contents were continuously increased in the leaf after 12 h of treatment with 25 and 100 mmol L^−1^ AlCl_3_ (Fig. 2b), and the increasing proline contents in the root was also observed after 72 h of treatment (Fig. 2b).

**Fig. 3:**
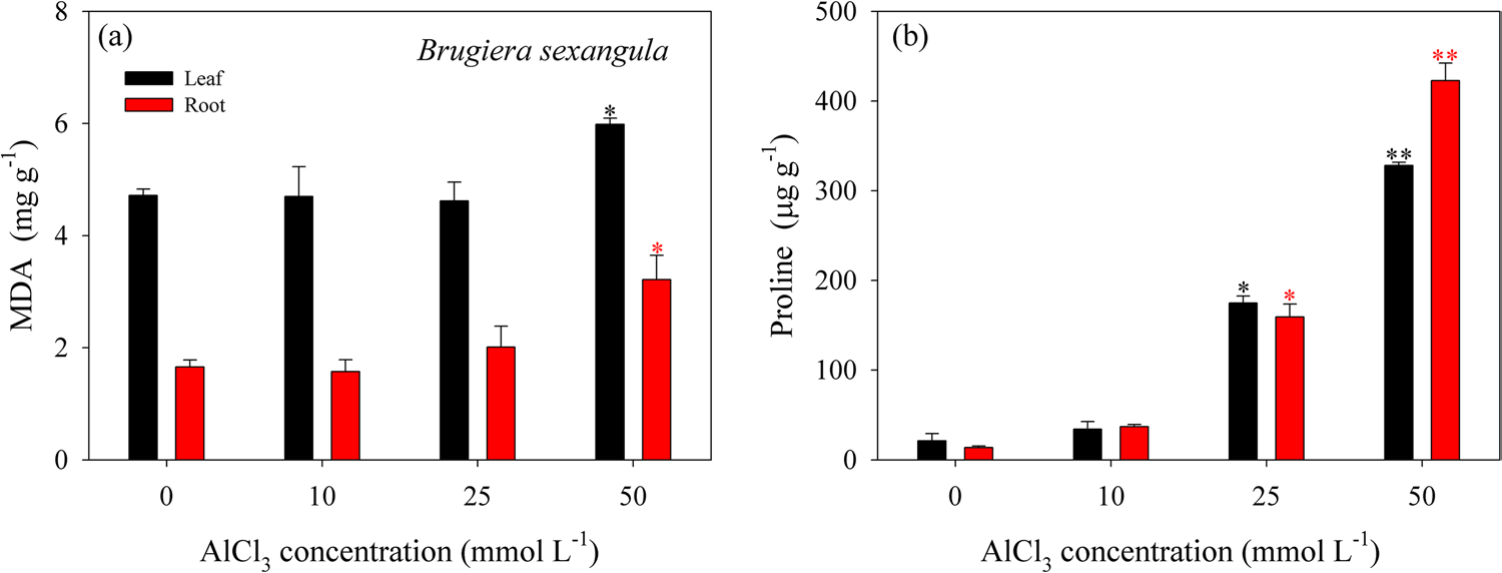
MDA content (**a**) and proline content (**b**) in the leaf (black bar) and in the root (red bar) of *B. sexangula* seedlings that under a long-term (60 days) treatment with 0–50 mmol L^−1^ AlCl_3_. Values are mean ± SD. “*” above the bar indicates a significant difference *versus* 0 mmol L^−1^ AlCl_3_ (control) at *P* < 0.05, and “**” above the bar indicates a significant difference *versus* 0 mmol L^−1^ AlCl_3_ (control) at *P* < 0.01

Both the MDA and proline contents in *B. sexangula* seedlings that under a long-term (60 days) treatment with 0–50 mmol L^−1^ AlCl_3_ are presented in Fig. 3. The MDA contents in *B. sexangula* seedlings were significantly increased in both the leaf and root under 50 mmol L^−1^ AlCl_3_ treatment (Fig. 3a), which was 1.3 and 1.9 times to that of control, respectively. The proline contents in both the leaf and root were also significantly increased in *B. sexangula* seedlings that were treated with 25 or 50 mmol L^−1^ AlCl_3_ (Fig. 3b), and the proline content was significantly higher under 50 mmol L^−1^ AlCl_3_ than that treated by 25 mmol L^−1^ AlCl_3_ (*P* < 0.01).

### Antioxidant enzyme activities in *K. obovata* and *B. sexangula* seedlings in response to aluminum

**Fig. 4:**
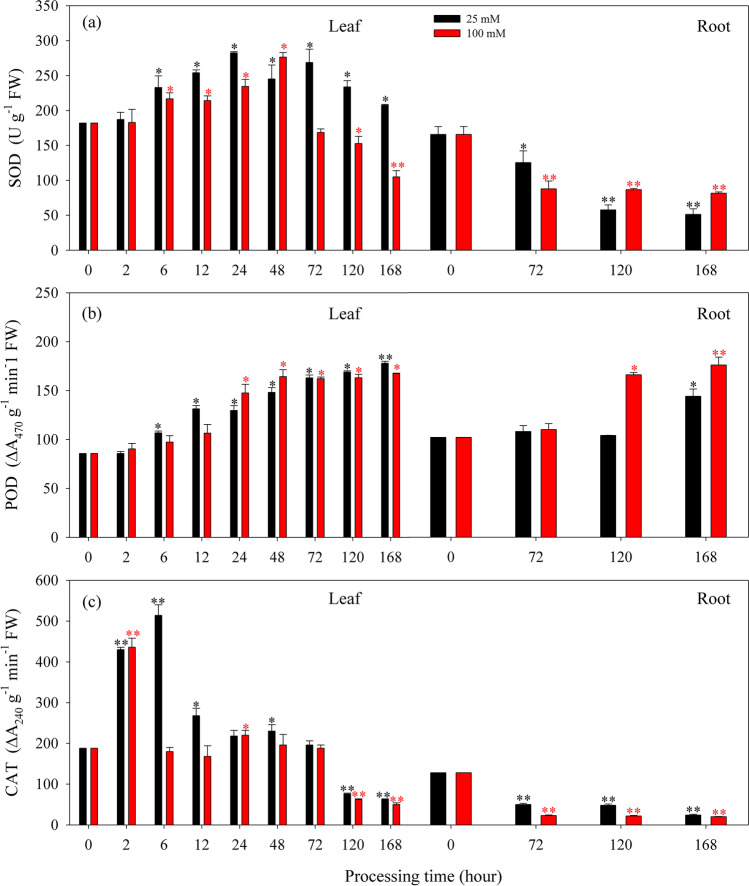
Changes in activities of SOD (**a**), POD (**b**), and CAT (**c**) in the leaf and root of *Kandelia obovata* seedlings that induced by short-term (168 h) AlCl_3_ treatments, 25 mol L^−1^ (25 mM, black bar) and 100 mol L^−1^ (100 mM, red bar). Values are mean ± SD. “*” above the bar indicates a significant difference *versus* 0 h group (control) at *P* < 0.05, and “**” above the bar indicates a significant difference *versus* 0 h group (control) at *P* < 0.01

The antioxidant enzyme activities in response to the oxidative stress that was induced by short-term (168 h) AlCl_3_ treatments in *K. obovata* seedlings are presented in Fig. 4. Results showed that antioxidant enzyme activities varied in *K. obovata* seedlings subjected to different AlCl_3_ treatments. SOD activity was increased after 6 h of 25 mmol L^−1^ AlCl_3_ treatment, with the maximum value observed after 24 h in the leaf (Fig. 4a), whereas for that of 100 mmol L^−1^ AlCl_3_ treatment, SOD activity was continuously decreased with a short period of increase after 6–48 h in the leaf (Fig. 4a). SOD activity in the root was significantly decreased after 72 h of 25 or 100 mmol L^−1^ AlCl_3_ treatment (Fig. 4a). Similarly, POD activity was significantly increased in the leaf after 6 h of 25 or 100 mmol L^−1^AlCl_3_ treatment (Fig. 4b), and POD activity in the root was significantly increased within 120 and 168 h after being treated with 100 and 25 mmol L^−1^ AlCl_3_, respectively (Fig. 4b). Overall, POD activity was higher under 100 mmol L^−1^ AlCl_3_ treatment than that under 25 mmol L^−1^ AlCl_3_ (*P* < 0.01). Furthermore, CAT activity was highly increased in the leaf after being treated by 25 or 100 mmol L^−1^ AlCl_3_ for 2 h, and decreased after 72 or 48 h, with the maximum value observed after 6 or 2 h, respectively (Fig. 4c). However, a significant decrease in CAT activity in the root was observed after 72 h of AlCl_3_ treatments (Fig. 4c), and CAT activity was higher under 25 mmol L^−1^ AlCl_3_ than that treated by 100 mmol L^−1^ AlCl_3_ (*P* < 0.05).

**Fig. 5:**
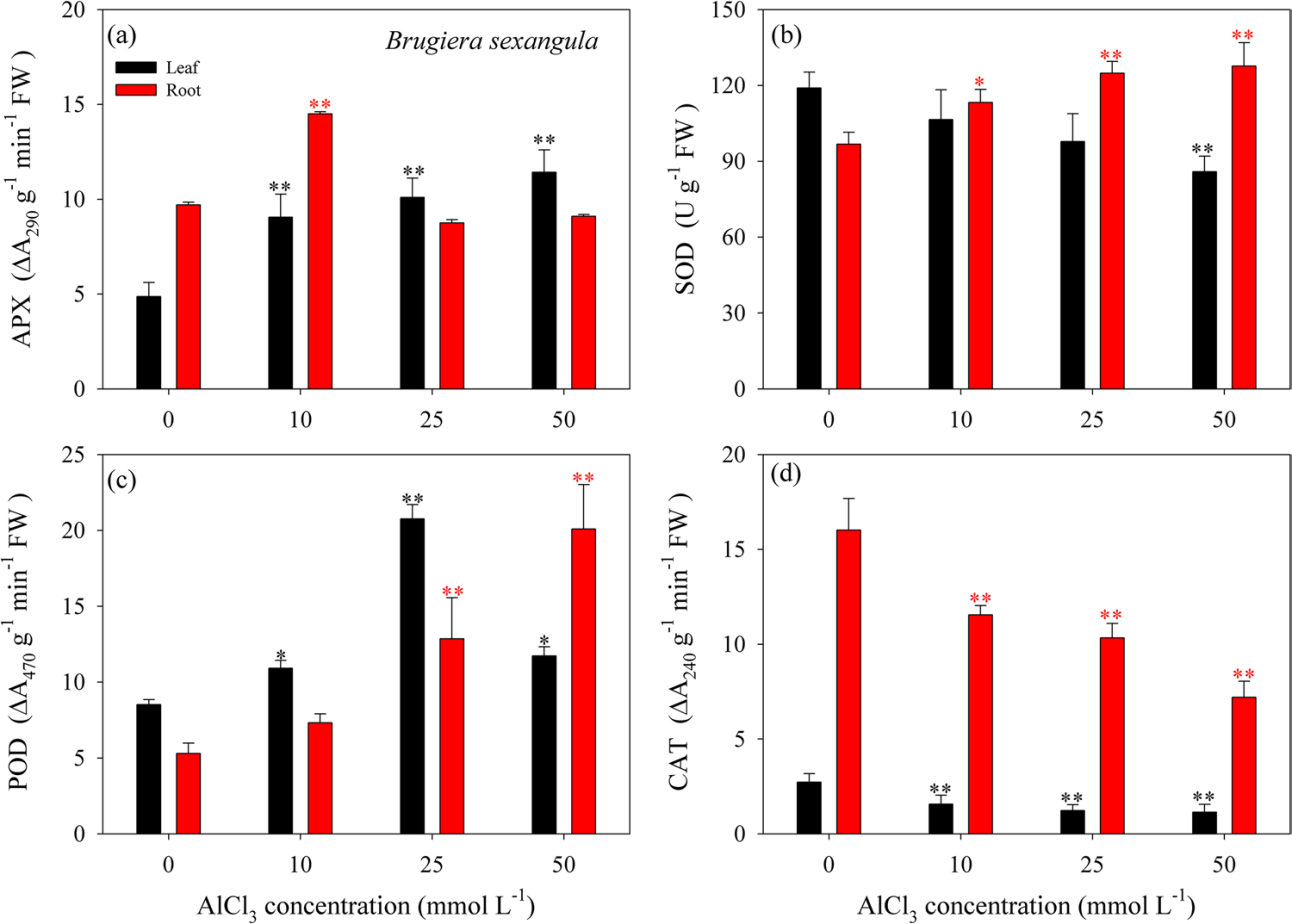
Changes in activities of APX (**a**), SOD (**b**), POD (**c**), and CAT (**d**) in the leaf (black bar) and root (red bar) of *B. sexangula* seedlings by long-term (60 days) treatment with 0–50.0 mmol L^−1^ AlCl_3_. Values are mean ± SD. “*” above the bar indicates a significant difference *versus* 0 mmol L^−1^ AlCl_3_ (control) at *P* < 0.05, and “**” above the bar indicates a significant difference *versus* 0 mmol L^−1^ AlCl_3_ (control) at *P* < 0.01

For *B. sexangula* seedlings obtained after a relative long-term (60 days) exposure to 10, 25, and 50 mmol L^−1^ AlCl_3_ treatments, APX activity was significantly increased in the leaf under 10–50 mmol L^−1^ AlCl_3_ treatment, whereas increased activity in the root was only observed under 10 mmol L^−1^ AlCl_3_ (Fig. 5a). SOD activity was increased in the leaf with the increasing AlCl_3_ concentrations (Fig. 5b); conversely, SOD activity was significantly decreased in the root under 50 mmol L^−1^ AlCl_3_ (Fig. 5b). Compared to that of control, POD activity was significantly increased in the leaf, with the maximum observed under 25 mmol L^−1^ AlCl_3_ (Fig. 5c). Additionally, a positive relationship between POD activity and AlCl_3_ concentration was observed in the root (*P* < 0.05) (Fig. 5c). In contrast, CAT activity was gradually decreased in both the leaf and root with the increasing AlCl_3_ concentrations (Fig. 5d). Notably, the activities of APX, SOD, and CAT were well correlated with MDA content in the leaf (*P* < 0.05), while SOD, POD, and CAT activities presented clear AlCl_3_ concentration–dependent relationships (*P* < 0.05).

### Metal element accumulation and translocation in *B. sexangula* seedlings

**Fig. 6:**
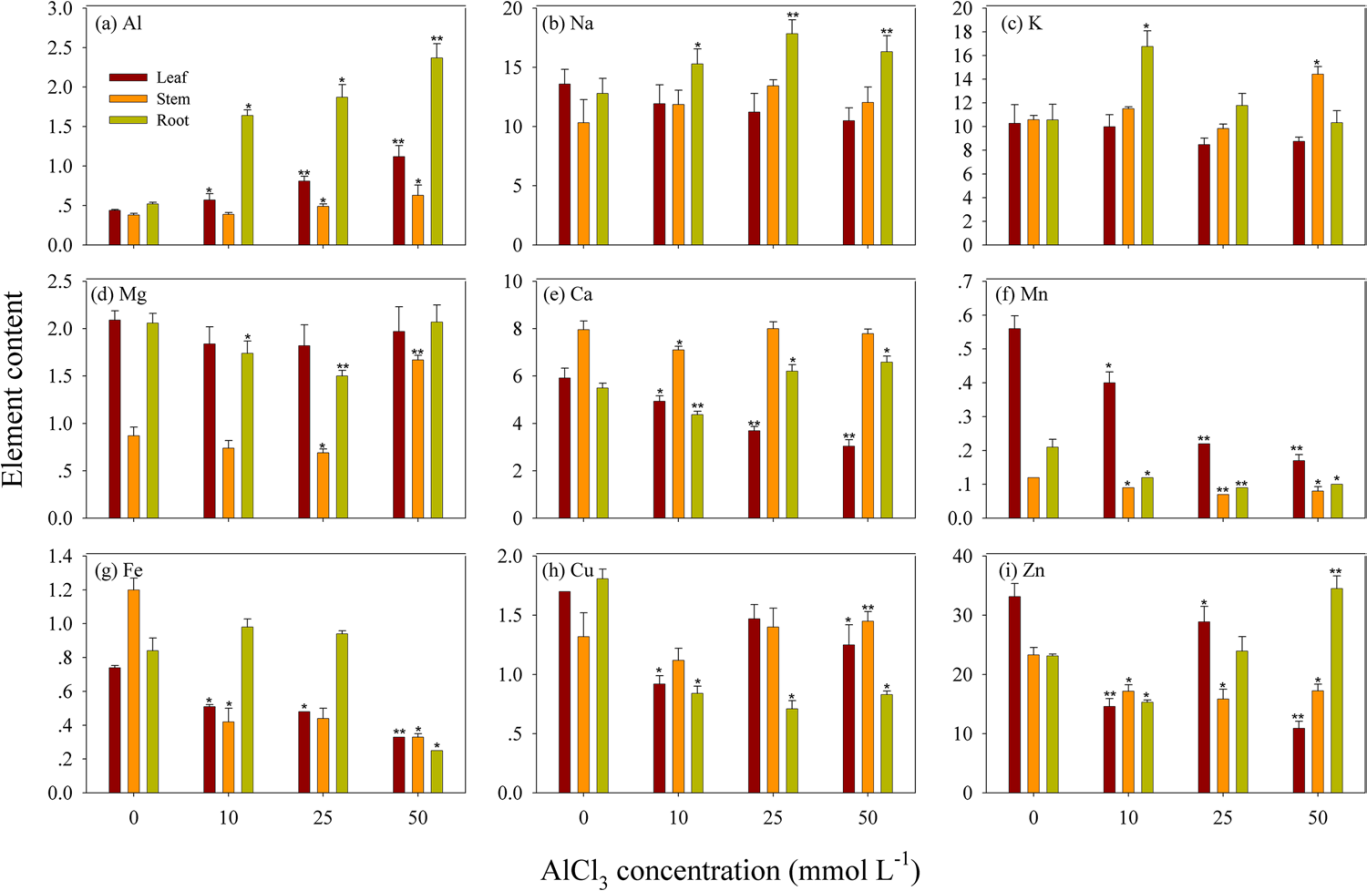
The element content of Al (**a**), Na (**b**), K (**c**), Mg (**d**), Ca (**e**), Mn (**f**), Fe (**g**), Cu (**h**), and Zn (**i**) in *B. sexangula* tissues (leaf, stem, and root) under a long-term (60 days) treatment with 0–50.0 mmol L^−1^ AlCl_3_. Note that the unit for (**a**–**f**) is (mg g^−1^), and unit for (**h**, **i**) is (μg g^−1^). Values are mean ± SD. “*” above the bar indicates a significant difference *versus* 0 mmol L^−1^ AlCl_3_ (control) at *P* < 0.05, and “**” above the bar indicates a significant difference *versus* 0 mmol L^−1^ AlCl_3_ (control) at *P* < 0.01

The contents of nine metal elements were generally different in various tissues of *B. sexangula* seedlings under AlCl_3_ treatments (Fig. 6). The contents of elements were aluminum (Al) of 0.38–2.37 mg g^−1^, sodium (Na) of 10.33–17.84 mg g^−1^, potassium (K) of 8.48–16.75 mg g^−1^, magnesium (Mg) of 0.69–2.09 mg g^−1^, calcium (Ca) of 3.04–7.96 mg g^−1^, manganese (Mn) of 0.08–0.56 mg g^−1^, iron (Fe) of 0.25–1.20 mg g^−1^, copper (Cu) of 0.71–1.81 μg g^−1^, and zinc (Zn) of 10.92–34.53 μg g^−1^.

Aluminum was significantly accumulated in all tissues of *B. sexangula* seedlings by all AlCl_3_ treatments; the accumulation pattern of Al was root > leaf > stem (Fig. 6a and Table 1). Na was also significantly accumulated in the root under all AlCl_3_ treatments (Fig. 6b), whereas K was only accumulated in the root under 10 mmol L^−1^ AlCl_3_, and in the stem under 50 mmol L^−1^ AlCl_3_ (Fig. 6c). The accumulation of Mg in the root was inhibited by 10–25 mmol L^−1^ AlCl_3_ treatment, whereas a significantly increase in Mg was observed in the stem treated by 50 mmol L^−1^ AlCl_3_ (Fig. 6d). The accumulation of Ca was significantly inhibited in the leaf, but increased in the root under 25–50 mmol L^−1^ AlCl_3_ (Fig. 6e). The accumulation of Mn was significantly inhibited in the leaf, stem, and root under 10–50 mmol L^−1^ AlCl_3_ (Fig. 6f). Similarly, the accumulation of Fe was decreased in both the leaf and stem under 10–25 mmol L^−1^ AlCl_3_, and a decrease to 70.1% was observed in the root under 50 mmol L^−1^ AlCl_3_ (Fig. 6g). A decrease in Cu accumulation in the leaf and root under AlCl_3_ treatments was also observed, but not in the stem (Fig. 6h). The accumulation of Zn was significantly decreased in the leaf and stem by all AlCl_3_ treatments; however, it was increased in the root under 50 mmol L^−1^ AlCl_3_ (Fig. 6i).

**Table 1:** The enrichment factors (EF) of Al, the ratio of Na/K and Al/Ca, and the translocation factors (TF) of metal element in *B. sexangula* seedlings under long term (60 days) of AlCl_3_ treatment

Al treatment (mmol L^−1^)	EF			Na/K			Al/Ca			TF								
	Leaf	Stem	Root	Leaf	Stem	Root	Leaf	Stem	Root	Al	Na	K	Mg	Ca	Mn	Fe	Cu	Zn
0				1.32	0.98	1.21	0.07	0.05	0.09	1.58	1.87	1.97	1.44	2.52	3.23	2.31	1.67	2.44
10	4.27	2.92	12.28	1.20	1.03	0.91	0.12	0.05	0.38	0.59	1.56	1.28	1.48	2.76	4.08	0.95	2.43	2.07
25	0.24	0.15	0.56	1.32	1.37	1.51	0.22	0.06	0.30	0.70	1.38	1.56	1.67	1.88	3.22	0.98	4.04	1.87
50	0.17	0.09	0.36	1.20	0.83	1.58	0.37	0.08	0.36	0.74	1.38	2.25	1.76	1.64	2.50	2.64	3.25	0.82

The EFs of AlCl_3_ in *B. sexangula* tissues (leaf, stem, and root) are presented in Table 1. The values of EFs in the leaf, stem, and root were significantly decreased, accompanied by increasing AlCl_3_ concentrations in the substrate. Under the same AlCl_3_ level, the highest EF of Al was observed in the root, whereas the lowest one was in the stem. The EFs of Al in each fraction of *B. sexangula* seedlings under 10 mmol L^−1^AlCl_3_ varied from 2.92 to 12.28, while those under other treatments were below the level of 1. For the ratio of Na/K, the lowest value was observed in the root after being treated with 10 mmol L^−1^ AlCl_3_ (Table 1), and Na/K was significantly increased with increasing AlCl_3_ concentrations in substrates. And similar result was also observed for the ratio of Al/Ca (Table 1).

**Fig. 7:**
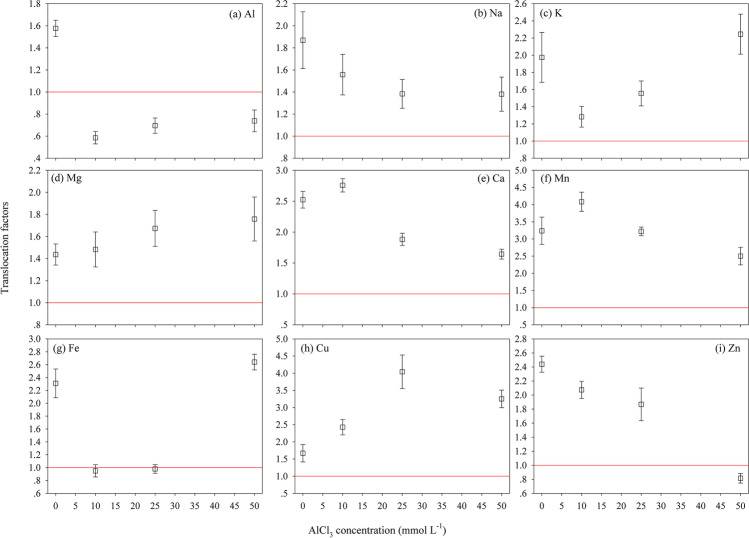
The translocation factor (TF) of Al (**a**), Na (**b**), K (**c**), Mg (**d**), Ca (**e**), Mn (**f**), Fe (**g**), Cu (**h**), and Zn (**i**) in *B. sexangula* seedlings under a long-term (60 days) treatment with 0–50.0 mmol L^−1^AlCl_3_. Values are mean ± SD

The TFs were defined as the ratio of the metal elements in the aboveground tissue (leaf and stem) to those in the underground tissue (root). The TF values of metal elements are presented in Fig. 7 and Table 1. Compared with the control, the TF values of Al were less than 1 under all AlCl_3_ treatments (Fig. 7a and Table 1). The TF values of essential metals in mangrove seedlings were differently affected by AlCl_3_ concentrations in the experimental media (Fig. 7b–i). For the essential metal elements of Na, K, Mg, Ca, Mn (Fig. 7b–f), and Cu (Fig. 7h), the TF values were often higher than 1, and Mg was the most translocated macro-nutrient that increased with the increasing AlCl_3_ concentrations (TF: 1.44–1.76). The TF values of Na (Fig. 7b), Ca (Fig. 7e), Mn (Fig. 7f), and Zn (Fig. 7i) were significantly decreased under AlCl_3_ treatments, particularly under a hyper AlCl_3_ concentration of 50 mol L^−1^. Notably, Mn and Cu were the most translocated trace metals (TF: 2.50–4.08 and 1.67–4.04, respectively).

## Discussion

### Aluminum stress tolerance in *K. obovata* and *B. sexangula* seedlings

*K. obovata* and *B. sexangula* differed in their ability to grow at varying AlCl_3_ concentrations. The two mangrove seedlings showed remarkable tolerance to hypo and hyper AlCl_3_ conditions, respectively. *K. obovata* survived in tested AlCl_3_ concentrations of 0.0625–10 mmol L^−1^, whereas that of *B. sexangula* was even higher with AlCl_3_ concentrations of 10–50 mmol L^−1^. In addition, *K. obovata* and *B. sexangula* seedlings had, respectively, high tolerance potential of 10 and 50 mmol L^−1^ AlCl_3_, suggesting that *B. sexangula* had a higher Al tolerance ability. Related studies have shown that the Al-resistance ability of plants depends on their species (Boscolo et al. 2003; Darkó et al. 2004). The tolerance of high concentrations of Al has been examined in some plants; for instance, soybean (*Glycine max* (L.) Merr.) seedlings can normally grow under 10–75 μmol L^−1^ Al stress (Cakmak and Horst 1991), tea plant (*Camellia sinensis* (L.) O. Ktze.) can tolerate 0.5–4.0 mmol L^−1^ Al treatments (Morita et al. 2008), and woody plants such as *Cryptomeria japonica* (L. f.) D. Don, *Pinus thunbergii* Parl., and *Populus tremula* (L.) native to acid soils have evolved various strategies to overcome Al stress (Brunner and Sperisen 2013). *K. obovata* and *B. sexangula* are both salt exclusion mangrove species belonging to Rhizophoraceae Pers., which is associated with heavy metal regulation (MacFarlane et al. 2007; Zhang et al. 2007; Dai et al. 2017). We proposed that mangroves can tolerate high concentrations of AlCl_3_ stress and hold more effective resistance to Al than other reported plants.

The effect of Al toxicity in plants is widely known to inhibit plant growth and root elongation under acidic conditions (Marschner 1991; Barceló and Poschenrieder 2002; Bertrand et al. 2002; Kochian et al. 2004; Sivaguru et al. 2003; Tamás et al. 2003; Fukuda et al. 2007). In our study, hypo and hyper AlCl_3_ adversely affected the root elongation and seedling growth in mangroves. Concerning the behavior of hypo AlCl_3_ (0.0625–1.0 mmol L^−1^) for *K. obovata*, the root elongation was inhibited compared with that of control (Fig. 1a). It was noticed that the seedling height of *B. sexangula* was significantly decreased in high AlCl_3_ conditions (25–50 mmol L^−1^) than in low ones (10 mmol L^−1^) (Fig. 1b). Rapid inhibition of root elongation of plant is the earliest visible symptom of Al toxicity (Kochian 1995; Sivaguru and Horst 1998; Yang et al. 2008; Panda et al. 2009; Kochian et al. 2015), which could occur within 1–2 h after exposure to Al (Ryan et al. 1993) and cause root stunting. These symptoms have been observed in *K. obovata* and *B. sexangula* seedlings: the root elongation was distinctly restrained, and the root tip was seriously damaged by AlCl_3_ stress.

### Effect of aluminum on lipid peroxidation and osmotic stress in *K. obovata* and *B. sexangula* seedlings

Metal stress generally increases oxidative damage and lipid peroxidation, thus promoting the accumulation of MDA in plants (Chaoui et al. 1997; Ozturk et al. 2010; Nahar et al. 2016; Dai et al. 2017). In this study, lipid peroxidation level (expressed as MDA content), as oxidative stress parameters, was significantly affected by AlCl_3_ in mangrove seedlings. When compared with the control, the contents of MDA were significantly increased in *K. obovata* seedlings under short-term (168 h) AlCl_3_ treatments at 25 and 100 mmol L^−1^ (Fig. 2a), and in *B. sexangula* seedlings under a long-term (60 days) AlCl_3_ treatment at 50 mmol L^−1^ (Fig. 3a). These experimental results support that *K. obovata* is damaged rapidly by ROS and it can be classified as a more stress-susceptible species. Although *B. sexangula* has a relatively high endogenous MDA content (control), MDA content was clearly increased in the both leaf and root after a long-term AlCl_3_ treatment at 50 mmol L^−1^ (Fig. 3a), suggesting that a high degree of lipid peroxidation occurred in *B. sexangula* seedlings, which can be considered as a more stress-tolerant mangrove species that could better avoid oxidative damage.

Proline accumulation under metal stress has been recognized as potential indicator of tolerance; the high levels of free proline accumulated in plants reflect their response to osmotic stress (Iyer and Caplan 1998; Ashraf and Foolad 2007; Ahmad et al. 2015). Increased production of proline directly indicates its protective role in scavenging free radical, stabilizing subcellular structure, and maintaining redox imbalance of the cell (Ahmad et al. 2015; Zouari et al. 2016). In this study, the contents of proline were significantly affected by AlCl_3_ in the two mangrove seedlings. For short-term AlCl_3_ treatments, *K. obovata* produced more proline with the elongation of the treatment time (Fig. 2b), suggesting the osmotic stress in *K. obovata* seedlings rapidly responded to AlCl_3_ stress. In contrast to *K. obovata*, the content of proline in *B. sexangula* seedlings was continuously maintained at a high level within 60 days (Fig. 3b), which was increased by 7.2–14.4 and 10.7–30.1 times in the leaf and root, respectively. These results explained well that *B. sexangula* has stronger and more effective resistance mechanisms than *K. obovata*. The accumulation of proline has been shown to positively correlate with the resistance of plants (Sharma and Dietz 2006). In the present study, with the increase of AlCl_3_ concentration, the content of proline was increased in the two mangrove seedlings, which may be attributed to the low external osmotic potential induced by Al stress. In order to avoid seepage damage, plant cells actively accumulate soluble substances, such as proline, to avoid damage caused by water loss (Zouari et al. 2016). Additionally, proline can combine with free metal ions such as Al^3+^ and form nontoxic Al-proline complexes, which can reduce Al toxicity to the active sites of enzymes (Sharma et al. 1998).

### Effect of aluminum on antioxidant enzyme activities in *K. obovata* and *B. sexangula* seedlings

Related studies have demonstrated that Al can induce severe oxidative stress in organisms, due to the formation of free radicals and disintegration of ROS metabolism by Al toxicity (Boscolo et al. 2003; Tamás et al. 2003; Darkó et al. 2004; Liu et al. 2008; de Sousa et al. 2016; Ali 2017; Awad et al. 2019; Chandra et al. 2020; Chandra and Keshavkant 2021). In response to Al stress, antioxidant enzymes, including SOD, CAT, POD, and APX, are correlated with the ability to scavenge ROS formed under stress conditions (Cakmak and Horst 1991; Tamás et al. 2003; Nahar et al. 2016; Al Mahmud et al. 2019; Devi et al. 2020). In the present study, both the two mangrove seedlings revealed significant changes in SOD, CAT, and POD activities throughout the entire range of AlCl_3_ concentrations. *K. obovata* and *B. sexangula* mostly depend on SOD as the first line of defense; SOD activity is directly related to stress by converting O_2_^−^ that drives cell damage to H_2_O_2_ (Hasanuzzaman et al. 2020). Then CAT and POD, as well as APX in chloroplasts, subsequently scavenge the generated H_2_O_2_ via a two-electron transfer producing O_2_ and H_2_O, thus avoiding the production of OH^−^ (Cakmak and Marschner 1992; Vranová et al. 2002; Nahar et al. 2016; Al Mahmud et al. 2019; Devi et al. 2020).

Evidently, the antioxidant enzyme activities are differently regulated by the treatment time and AlCl_3_ concentration between mangrove species. It is likely that, SOD activity in *K. obovata* increased for dismutation of superoxide ion and the generated H_2_O_2_ was subsequently degraded by CAT and POD within 168 h (Fig. 4a–c). Whereas the activity of APX and SOD, respectively, increased in the leaf and root of *B. sexangula* seedlings, the generated H_2_O_2_ was subsequently degraded by POD after exposure to AlCl_3_ treatments at extreme concentrations within 60 days (Fig. 5a–c). Increases in antioxidant enzyme activities are known as the primary prevention of stress damage, and elevated activities of SOD and POD might result from the de novo synthesis and expression of enzymatic proteins induced by Al stress (Cakmak and Horst 1991; Devi et al. 2003; Sivaguru et al. 2003). Additionally, the function of POD has been widely accepted as a potential biomarker for sub-lethal metal toxicity in plants (Radotić et al. 2000). High levels of POD activity observed in *K. obovata* and *B. sexangula* seedlings suggested a better intrinsic defense to resist Al-induced oxidative damage in mangroves. Notably, APX is an important enzyme for decomposing H_2_O_2_ and O_2_^−^ through the ascorbic acid-glutathione cycle in the chloroplast, and the increase in APX activity can be considered the defensive response to ROS (Cakmak and Marschner 1992). In the leaf of *B. sexangula* seedlings, APX activity increased and compensated for the decline of CAT activity (Fig. 5 a and d), suggesting that APX is involved in the degradation of H_2_O_2_ generated by Al stress.

### Effect of aluminum on metal accumulation and translocation in *B. sexangula* seedlings

From the study of long-term (60 days) AlCl_3_ treatments on *B. sexangula* seedlings, the order of Al accumulation was root > leaf > stem (Fig. 6a and Table 1), and the uptake ability of Al with the highest EF value was observed in the root (Table 1), suggesting that root is the primarily important tissue of mangrove seedlings for Al deposition. The varying EFs of Al among tissues of *B. sexangula* seedlings after being treated with different concentrations of AlCl_3_ (Fig. 6a and Table 1) also indicated that *B. sexangula* differed in its absorption ability and accumulation response to different levels of Al stress. The TF values of Al were less than 1 under all AlCl_3_ treatments (Fig. 7a and Table 1), suggesting that the root can accumulate Al and limit its translocation to the leaf and stem to prevent Al toxicity.

Macro metal elements such as Na, K, and Mg are essential for the mangroves (Reef et al. 2010; Alongi 2018). Essential macro-elements like Na showed hyper-accumulation in the root under all AlCl_3_ treatments, being highest at 25 mmol L^−1^ AlCl_3_ (Fig. 6b). The distribution patterns of Na and K (Fig. 6 b and c) indicated that the root is the major tissue for the accumulation of these two metals. The ratio of Na/K increased with the increasing AlCl_3_ concentrations in substrates (Table 1), suggesting the distinctly selective absorption of Na by the root and upward transport to the leaf. Conversely, the accumulation of Mg in the root was inhibited under 10–25 mmol L^−1^ AlCl_3_ treatment (Fig. 6d), whereas the increased TF values of Mg (1.44–1.76) under AlCl_3_ treatment suggested that its translocation from the root to the aboveground tissues is efficient. These results suggested that the root is a very sensitive tissue that is affected by AlCl_3_ stress, whereas Al has weak impact on the transport of Na, K, and Mg in *B. sexangula* seedlings. We proposed that *B. sexangula* seedlings might hold a positive adaptation mechanism to Al stress, while stable levels of Na, K, and Mg in the leaf could benefit to maintain their normal metabolic processes, such as photosynthesis and respiration.

Calcium plays an important role in maintaining the stability of cell wall and membrane, and regulating the balance of ions and antioxidant enzymes (Cakmak and Horst 1991). Related studies have proposed that interactions between Al and Ca are probably the most important factors affecting Ca uptake and transport in plants. Al stress could disturb the absorption balance of calcium cation (Ca^2+^) in plant cells, which further induced the generation of superoxide (Huang et al. 1992; Kawano et al. 2004). In the present study, the increase in Ca accumulation in the root might be attributed to an important mechanism in which seedlings employ the aggravating absorption of Ca in the root to relieve Al toxicity. However, the ratio of Al/Ca was increased with the increasing AlCl_3_ concentration in the substrate (Table 1), suggesting that the effect of Ca on the suppression of Al toxicity might be restrained by high concentrations of AlCl_3_.

The uptake of micronutrients is primarily controlled by plant metabolic requirements, resulting in variable metal accumulations in different mangrove tissues. It is well known that Mn and Fe are essential nutritional elements for plant growth, particularly as an electron carrier in metabolism, protein synthesis, photosynthesis, chloroplast development, and antioxidant enzyme activities (Bertrand et al. 2002; Bose et al. 2014; Hasanuzzaman et al. 2020). Lack of Mn or Fe would cause protein reduction in the reaction center of photosynthetic system II (Green et al. 1991). Our results suggested that Al stress inhibited the absorption and transport of Mn and Fe in *B. sexangula* seedlings (Fig. 6 f and g), which might further impact the physiological and biochemical processes that regulate functional enzyme activities. As an essential trace metal element for plants, Cu is closely involved in chlorophyll formation, as well as synthesis of proteins (Uriu-Adams and Keen 2005). Notably, in this study, the seedling curled leaf with brown lesions and the black root tips, suggesting that the accumulation and transport of Cu have been inhibited by Al stress. Zn is an essential catalytic component of over 300 enzymes, which could protect the sulfhydryl groups of enzymes against free radical attack, and it acts as an antagonist against redox-active transition metals including Fe and Cu (Frassinetti et al. 2006). The increase in Zn in the root under hyper AlCl_3_ conditions (Fig. 6i) suggested its positive response to Al stress, compared to the opposite accumulation patterns of Fe and Cu in roots.

Mangrove plants accumulate metal micronutrients in the aboveground tissues (stem and leaf) because some metals are required for their growth and survival (Huang et al. 2020). For the essential metal elements of Na, K, Mg, Ca, Mn, and Cu, their TF values were often higher than 1 (Table 1), suggesting that the translocation of these metals from the root to the aboveground tissues is efficient. Conversely, the TF value of Zn was less than 1 under 50 mmol L^−1^ AlCl_3_ treatment (Table 1), indicating an inhibition of translocation of this micronutrient. Notably, the TF values of Na, Ca, Mn, and Zn were significantly decreased with the increasing AlCl_3_ concentration in the substrate (Fig. 7 b, e, f, and i), particularly under hyper AlCl_3_ of 50 mmol L^−1^, suggesting that the translocation of these elements is inhibited by Al stress. In this study, Mn and Cu are the most translocated trace metals in mangrove issues, which are expected to play important roles in overall plant physiology (e.g., photosynthesis, chloroplast development, and sustenance of metabolism) (Reef et al. 2010; Bose et al. 2014; Alongi 2018; Hasanuzzaman et al. 2020; Huang et al. 2020). The differences between TFs of trace metals in mangroves may not necessarily be linked to the translocation of elements (Huang et al. 2020). *B. sexangula* was shown to have the highest TF values of Cu (up to 4.08) and Mn (up to 4.04), indicating that the translocation of these metals from the root to the shoot of this species efficiently occurs under Al toxicity. Typically, mangroves exhibit high TF values of essential elements such as Mn and Cu (MacFarlane et al. 2007).

These results highlight that the accumulation and translocation of metal elements are mainly inhibited in mangrove seedlings, in response to AlCl_3_ stress. To some extent, the tolerance of mangroves to heavy metals depends on the elimination and adjustment of heavy metals and the restriction of heavy metal translocation from the underground to aboveground tissues (Wang et al. 2016). In this study, although the accumulation and translocation of eight essential metal elements are differently affected by Al stress, the root is the most sensitive tissue for metal enrichment. The plants that have a certain degree of tolerance to polluted surroundings usually store heavy metals in the vacuole of the root cortex tissue or cell wall, so they can reduce heavy metal concentrations in the aboveground tissues (Zhao et al. 2015). Furthermore, metal transfer in a root-stem-leaf order can be affected by leaf transpiration; transpiration acts as a controller in Al transport (Liu et al. 2016), which has been confirmed in several plants (Van der Vliet et al. 2007; Liu et al. 2010).

## Conclusions

This work demonstrates that the mangroves *K. obovata* and *B. sexangula* can adapt to high levels of AlCl_3_ in acid conditions, with the tolerance to Al toxicity reaching a maximum concentration of 10 and 50 mmol L^−1^, respectively. Mangrove seedlings can grow and survive in high Al environments, but with reduced root length and seedling weight as the negative response to Al stress. Under AlCl_3_ treatments, two features are evident in this study: (1) Al stress increased lipid peroxidation (expressed as MDA content) and osmotic pressure (indicated in proline content) levels, suggesting ROS molecules destroy membrane lipids. Antioxidant enzymes were activated under hyper AlCl_3_ conditions; changes in enzyme activities indicate that oxidative stress is induced. Synergistic effects of SOD, CAT, POD, and/or APX could maintain the metabolic balance of active oxygen in *K. obovata* and *B. sexangula* seedlings within a certain time. As a general tolerance mechanism, the growth behavior of mangrove seedlings under Al stress is correlated with the elevated activities of protective enzymes. (2) In response to Al stress, *B. sexangula* seedlings primarily retained Al in the root, and the low TF values of Al in the aboveground tissues (stem and leaf) support that low mobility is used as a strategy to avoid excessive uptake of metals into seedling bodies. The different tolerant abilities of mangrove species provide valuable information for mangrove rehabilitation in acid conditions and their adaptation to Al stress. To provide a better insight into the effect of geochemical conditions on the survival and growth of mangroves, further field studies should be extended to monitor the responses of mangrove species to continuously long exposure to Al.

## Data Availability

All data can be accessed in the form of Excel spreadsheets via the corresponding author.
